# A new subspecies of African fire salamander *Salamandra algira* (Urodela, Salamandridae) from the Middle Atlas Mountains, Morocco

**DOI:** 10.3897/zookeys.893.46649

**Published:** 2019-12-02

**Authors:** Axel Hernandez, Daniel Escoriza

**Affiliations:** 1 Department of Environmental Sciences, Faculty of Sciences and Technics, University Pasquale Paoli of Corsica, Corte, 20250, France University Pasquale Paoli of Corsica Corte France; 2 GRECO, Institute of Aquatic Ecology, University of Girona, Girona, 17071, Spain University of Girona Girona Spain

**Keywords:** Amphibia, mitochondrial DNA, *Salamandra algira atlantica* ssp. nov., taxonomy, threatened species

## Abstract

A new subspecies within the *Salamandra algira* species complex from north-western Africa is described. Previous molecular analysis showed that the populations of *S. algira splendens* from north-western Morocco consisted of two well supported clades: clade 1 distributed in the Rif Mountains, from Chefchaouen (type locality) to Al Hoceima, and clade 2, located southern from clade 1 being isolated in the northern and central Middle Atlas Mountains. Clade 2 is herein described as a distinct subspecies: *Salamandra algira atlantica***ssp. nov.** based on morphological data, allopatric range and molecular divergence. This new subspecies shows an uncorrected pairwise distance of 0.0265 from clade 1 based on cytochrome b DNA sequences. *Salamandra algira atlantica***ssp. nov.** is a slender and large sized salamander with a highly variable colouration pattern. It can be distinguished from *S. algira splendens* by the greater proportion of coral red in the background colouration, being the only known subspecies of *S. algira* in which coral red can exceed the proportion of black. Variable number (0–5) of yellow to golden yellow dorsal blotches, but usually in lower numbers than the nominotypical subspecies. *Salamandra algira atlantica***ssp. nov.** inhabits subhumid to humid forests and karstic systems at mid to high elevations. We briefly discuss the phylogenetic and taxonomic issues among the genus *Salamandra* which encompasses more valid species than currently recognised.

## Introduction

The batrachofauna of North Africa was considered species-poor and mostly composed of species closely related to the European counterparts. However, several recent studies revealed a high degree of endemism and genotypic divergence (Recuero et al. 2007; Escoriza and Ben Hassine 2019). The genus *Salamandra* Garsault 1764 is a group of terrestrial Urodeles widely distributed across the western Palaearctic, comprising six species: *S. algira* Bedriaga, 1883, *S. atra* Laurenti, 1768, *S. corsica* Savi, 1838, *S. infraimmaculata* Martens, 1885, *S. lanzai* Nascetti, Andreone, Capula & Bullini, 1988 and *S. salamandra* (Linnaeus, 1758) (Dubois and Raffaëlli 2009). The genus is particularly well diversified through the Iberian Peninsula, possibly caused by several events of allopatric speciation during the Plio-Pleistocene climatic cycles (Steinfartz et al. 2000). In North Africa, *S. algira* is the sole representative of the genus (Escoriza and Ben Hassine 2019). The ancestor of *S. algira* possibly colonised the African continent during the Miocene from the Iberian Peninsula (Escoriza et al. 2006; Beukema et al. 2010). Later climatic fluctuations during the Pliocene and Pleistocene have driven allopatric genetic divergence in *S. algira* populations across the mountainous systems of northern Morocco and Algeria (Beukema et al. 2010; Ben Hassine et al. 2016).

*Salamandra algira* shows a fragmented distribution, being mostly confined to humid habitats in mountain ranges between elevations of 30–2455 m above sea level (a.s.l.) (Escoriza and Ben Hassine 2015). Four subspecies are currently recognised (Donaire-Barroso and Bogaerts 2003; Escoriza and Comas 2007; Beukema et al. 2013): *S. algira tingitana* in the north-western Rif, ranging from Ceuta to Moulay Abdessalam; *S. algira splendens*, in the central-eastern Rif, from Chefchaouen to Al Hoceima, including isolated populations in the Middle Atlas Mountains; *S. algira spelaea* restricted to the Beni Snassen massif; and the nominal *S. algira algira* in Algeria, located across the pericoastal mountains of Annaba, Collo, Kabylia, and Blida Atlas (Beukema et al. 2010; Escoriza and Ben Hassine 2014; Hernandez and Escoriza 2017). However, recent phylogenetic analysis suggested that the taxonomy of *S. algira* could require further re-assessments (Ben Hassine et al. 2016; Dinis et al. 2019). *Salamandra algira splendens* includes two distinct allopatric clades separated by 50 km of semi-arid plains. Here, we described one of these clades as a distinct subspecies, based on genetic and morphological evidences.

## Materials and methods

### Field sampling

Voucher specimens (i.e., the three specimens that constituted the type series) were anesthetised and euthanised in a closed vessel with a piece of cotton wool containing ethyl acetate (Simmons 2002), fixed in 95% ethanol for five hours, and subsequently transferred to 70% ethanol for permanent storage. Type specimens are held at the collections of vertebrates of the Museum of Natural Sciences of Barcelona (MZB) and Madrid (MNCN), Spain. Other specimens (19 adults and one larva) used in this study were measured, photographed and released in situ.

### Morphological examination

All specimens were sexed based on the cloaca morphology, because this is a constant sexual dimorphic character for species of the genus *Salamandra* (Raffaëlli 2013). The morphological comparison between the new taxon and other subspecies were based on voucher specimens, specimens captured in situ (Middle Atlas) and the original descriptions: Donaire-Barroso and Bogaerts (2003), Escoriza and Comas (2007) and Beukema et al. (2013).

We measured 14 morphological characters for adult and larval specimens, using a digital calliper (accuracy 0.01 mm) following Escoriza and Ben Hassine (2019). For adults, the abbreviations are:

**TL** total length;

**SVL** snout-cloaca length;

**HL** head length;

**PAL** parotoid length;

**HW** head width;

**IOR** interorbital distance;

**ED** eye diameter;

**END** eye nostril distance;

**LHU** humerus length;

**FAL** forearm length;

**HAL** hand length;

**THL** thigh length;

**TIL** tibia length;

**FL** foot length.

For larva the following abbreviations are used:

**SVL** snout-cloaca length;

**TAL** tail length;

**HL** head length;

**HW** head width;

**TL** total length;

**MTH** maximum tail height.

TL has been compared between the sexes of the new subspecies using a Mann-Whitney U test, conducted with Statistica 7.0 (StatSoft, Tulsa, OK).

### Molecular analysis

We inferred a molecular phylogeny based on sequence data from GenBank. Sequences have been obtained for much of the geographical range occupied by *S. algira*, including all major clades (Ben Hassine et al. 2016; Dinis et al. 2019). We aligned the downloaded sequences using ClustalW2 (Thompson et al. 1994). The final alignment included 309 base pairs of cytochrome b DNA partial genes of 106 specimens. The matrix of pairwise uncorrected p-distances was constructed using MEGA-X 10.0.5, estimating the variation based on 1000 bootstraps (Kumar et al. 2018). The phylogenetic analyses were performed under the Bayesian context using MrBayes v.3.2.5 (Huelsenbeck and Ronquist 2001). The best model of DNA substitution was selected using ModelTest 3.7 (Posada and Crandall 1998). We used the general-time reversible + invariant + gamma (GTR+I+G) substitution model, with 20 million generations of Monte Carlo Markov chains (MCMC), sampling every 10000 and discarding the first 25% of the trees (Huelsenbeck and Ronquist 2001). The convergence of MCMC simulations was determined checking the values of the effective sample sizes for the posterior probability, which was higher than 1000 for all continuous parameters (Ronquist et al. 2012). The phylogenetic tree was generated by iTOL 4.4.2 (Letunic and Bork 2006).

## Results

### Molecular analyses

**Figure 1.**
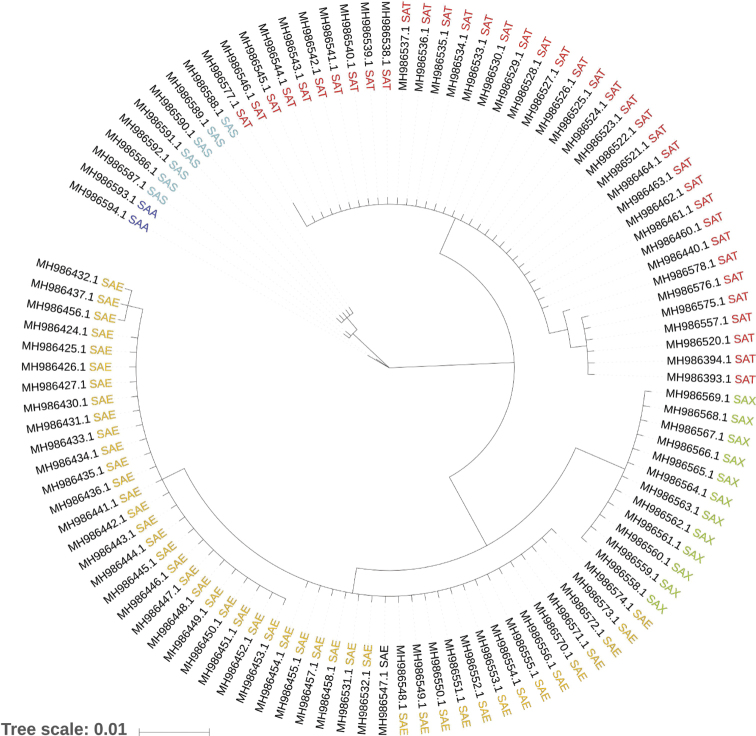
Phylogenetic relationships among *Salamandra algira* subspecies, inferred from cytochrome b sequences. Abbreviations: SAA = *S. algira algira* (dark blue); SAS = *S. algira spelaea* (sky blue); SAX = *S. algira atlantica* ssp. nov. (lime green); SAE = *S. algira splendens* (orange); SAT = *S. algira tingitana* (red).

Mean uncorrected p-distance between *S. algira* from the Atlas Mountains and *S. algira splendens* is 0.0265 ± 0.0082, similar to the divergence between the eastern subspecies of *S. algira* (*S. algira algira* and *S. algira spelaea*) (Table 1). The phylogenetic tree showed two well supported clades (Bayesian posterior probability, PP = 1.00) (Fig. 1) within the formerly taxonomic unit *S. algira splendens*: one belonging to the northern clade of the Rif Mountains, *S. algira splendens* sensu stricto, and the other one, distributed in the Middle Atlas Mountains, which is herein described as a new subspecies. Therefore, the western Moroccan group of *S. algira* is composed by three main morphologically diagnosable mitochondrial clades having subspecific status: *S. algira tingitana*, *S. algira splendens* and *S. algira atlantica* ssp. nov.

**Table 1.** Mean uncorrected *p*-distances based on isolate cytochrome b DNA sequences.

	*S. algira algira*	*S. algira spelaea*	*S. algira tingitana*	*S. algira splendens*
*S. algira algira*				
*S. algira spelaea*	0.0233			
*S. algira tingitana*	0.0573	0.0704		
*S. algira splendens*	0.0772	0.0756	0.0440	
*S. algira atlantica*	0.0777	0.0735	0.0518	0.0265

### Taxonomy

#### Salamandra algira atlantica ssp. nov.

Taxon classificationAnimaliaCaudataSalamandridae

9B8AC4DB-A27E-5A23-B71E-AB0BA293A4A3

http://zoobank.org/22C2B256-0F9B-4063-93B4-7C3DB84AA9E4

**Figure 2.**
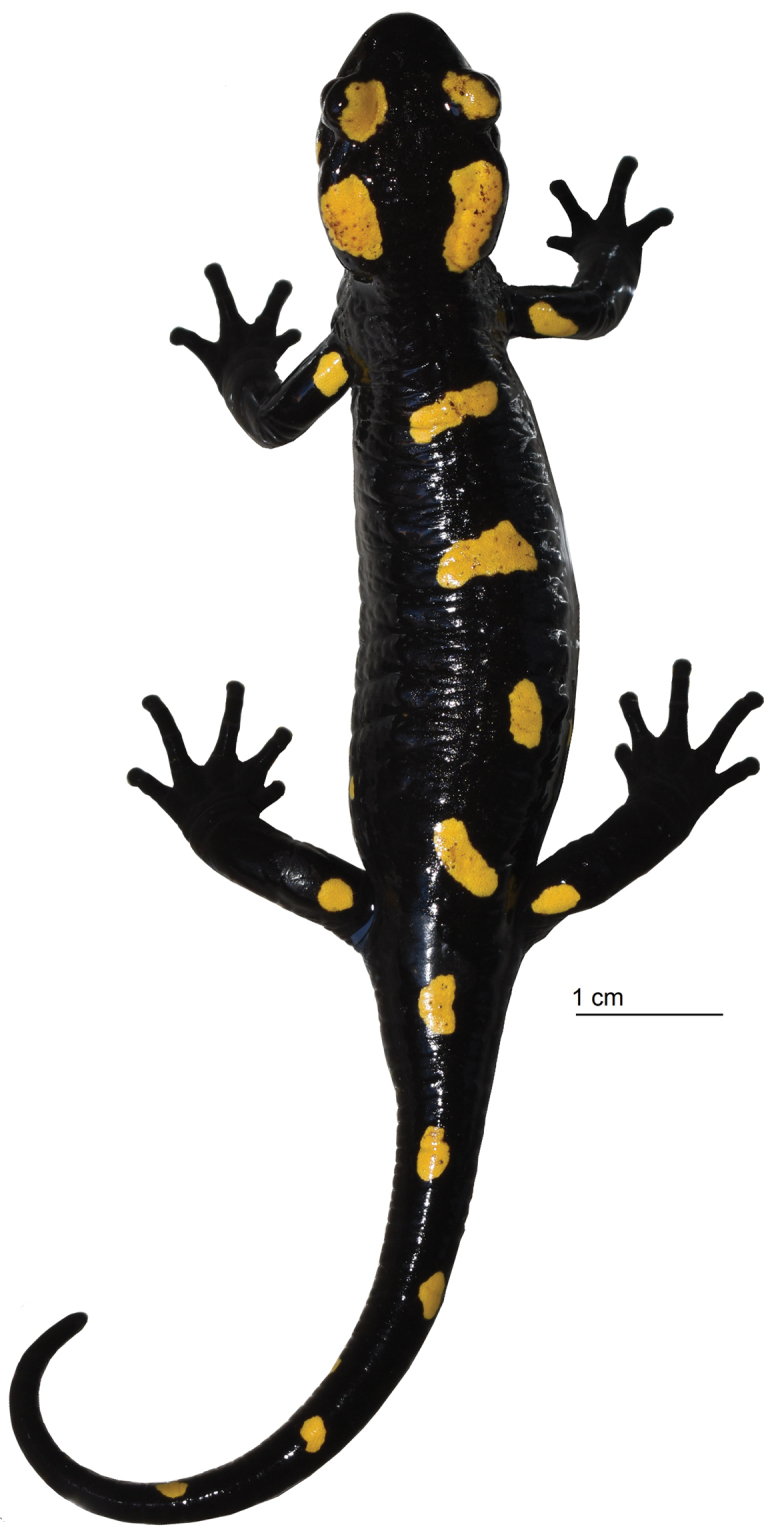
Holotype of *Salamandra algira atlantica* ssp. nov. (MNCN 50499) in life. Photograph by Daniel Escoriza.

**Figure 3.**
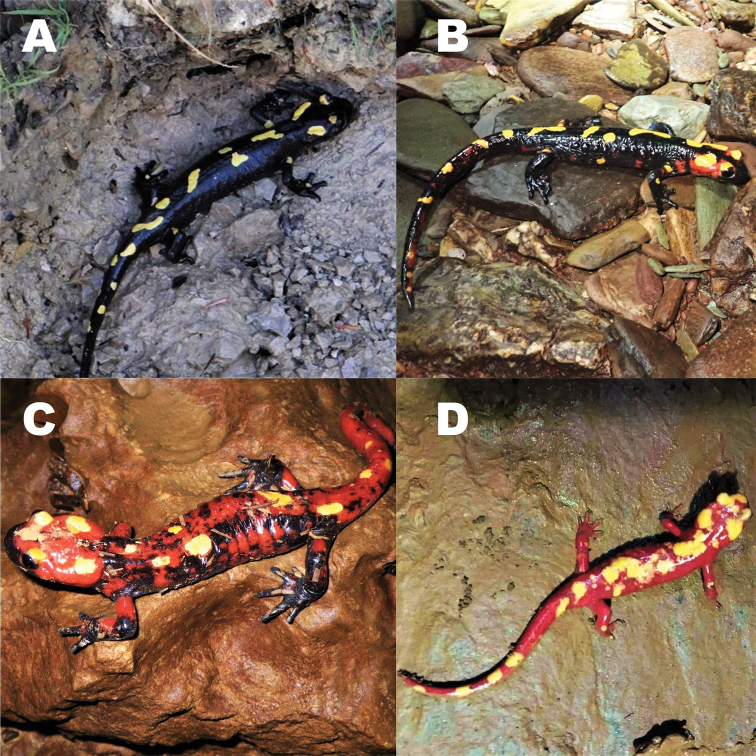
Variation in the colouration pattern of *Salamandra algira atlantica* ssp. nov.: **A** adult male from Jbel Sidi Ali, Midelt Province **B** adult female from Grotte de Chaâra, Taza Province **C, D** adult males from Grotte de Chaâra. Photographs by Axel Hernandez.

**Figure 4.**
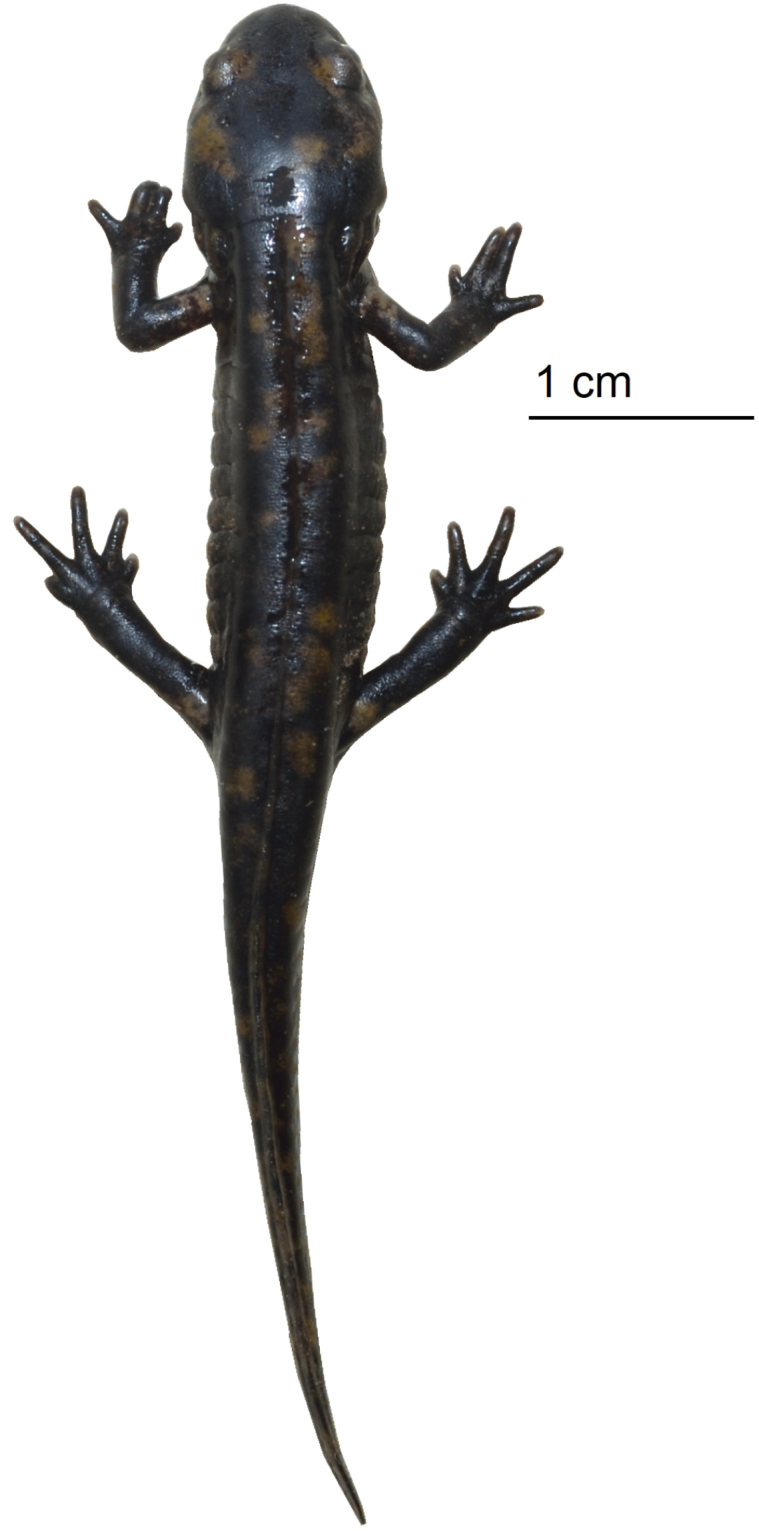
Larva of *Salamandra algira atlantica* ssp. nov. Photograph by Daniel Escoriza.

Figs 2 , 3 , 4 Atlas fire salamander

##### Holotype.

MNCN 50499 (Fig. 2), an adult male preserved in 70% ethyl alcohol from Jbel Tazekka, Taza Province, Middle Atlas Mountains, northern Morocco (34.15N, 4.00W) at 810 m a.s.l collected on 25 March 2013 by Daniel Escoriza.

##### Paratypes.

MZB 2010-0128 and MZB 2010-0129, two adult males preserved in 70% ethyl alcohol collected in the Jbel Tazekka, Taza Province, Middle Atlas Mountains, northern Morocco in December 2009 by Daniel Escoriza and Félix Amat.

##### Etymology.

The new taxon is named after the word ‘*Atlas*’ from Greek Aτλας in reference to the Atlas Mountains (Morocco), where this subspecies is found.

##### English name.

Atlas fire salamander.

##### Diagnosis.

A large subspecies of *S. algira* with a maximum total length of 246.21 mm. Distinguished from the other subspecies by the following combination of characters (Table 2):

**Table 2.** Identification key to *Salamandra algira* subspecies.

	*Salamandra algira algira*	*Salamandra algira spelaea*	*Salamandra algira tingitana*	*Salamandra algira splendens*	*Salamandra algira atlantica* ssp. nov.
Total length (in mm)	131–192 ♂ 129–255 ♀	177–194 ♂ 206–236 ♀	151–170 ♂ 140–192 ♀	140–178 ♂ 161–261 ♀	125–246 ♂ 133–205 ♀
Background colouration	Grey-black	Grey-black	Grey-black	Grey-black or coral red	Grey-black or coral red
Number of dorsal yellow blotches	3–10	3–10	0–7	2–7	0–7
Red spots	Usually small	Small	Absent	Small to large	Small to full
White spots on flanks	Present	Present	Absent	Absent	Absent

Background dorsal pattern variates from full grey-black to full coral red, being the only subspecies of *S. algira* in which the coral red can exceed the proportion of grey-black. *Salamandra algira algira* and *S. algira spelaea* usually show only few and little red colouration, mostly around yellow blotches on the dorsum of head, limbs and tail and gular region. *Salamandra algira tingitana* typically lacks red colouration. *Salamandra algira splendens* never shows full red background colouration, being mainly limited to the edges of the yellow blotches, on the dorsum of the head, limbs, tail and gular region.

Variable number (0–7, usually 2–4) of yellow to golden yellow dorsal blotches, but in lower numbers than the eastern subspecies (*S. algira algira* and *S. algira spelaea*, 3–10). *Salamandra algira tingitana* can be completely black or with numerous fragmented little yellow spots or with few large blotches in an arrangement similar to *S. algira atlantica* ssp. nov. *Salamandra algira splendens* has a similar number of yellow blotches than *S. algira atlantica* ssp. nov., but these can be more irregular in their shape in the former. Moreover in *S. algira atlantica* ssp. nov. dorsal yellow blotches can fade progressively into white, composing a pattern that does not appear in *S. algira splendens* (Fig. 3D).

Absence of white spots in the flanks of the body, which appear in the nominotypic subspecies and *S. algira spelaea*.

##### Description of holotype.

Snout rounded in a dorsal aspect and in lateral profile. Head large and well differentiated from the body. Nostrils oriented dorsolaterally, situated close to the snout tip. Large-sized parotoids, elongatedly ovoid, slightly divergent anteriorly, showing contrasted glandular pores. Prominent eyes situated laterally, with barely visible vertical oval pupils. Gular fold present. Costal grooves 10. Fingers short and slightly rounded, with a relative length I < IV < II < III. Toes slightly rounded, with a relative length I < V < II < III < IV. Cloaca ovoid. Subcylindrical tail, ended in a blunt tip. Dorsal skin weakly granular. Two pairs of glandular pores disposed in parallel, along the vertebral axis.

##### Colour of holotype.

In life the background dorsal colouration is black with four elongated golden yellow spots. In the head appear two golden yellow spots on the orbits and two on the parotoids. Four yellow spots at the base of the limbs and five on the dorsum of the tail. Two yellow spots in lateral parts of the body, close to the insertion of the hindlimbs. One small yellow spot showing red colouration in lateral part of the head, located posterior and ventral to the orbit. Uniform greyish black ventral colouration. Eyes dark brown, almost black.

##### Measurements of holotype (in mm).

TL 175.19 mm, SVL 99.16 mm, HL 20.82 mm, PAL 9.89 mm, HW 14.57 mm, IOR 6.17 mm, ED 5.05, END 4.81 mm, LHU 8.01 mm, FAL 11.52 mm, HAL 13.07 mm, THL 6.53 mm, TIL 11.93 mm, FL 14.8 mm.

##### Variation.

There are intraspecific population variations regarding colouration pattern in adult specimens (Fig. 3). Within the same population, yellow spots on the dorsum of the head can be divided into four semisymmetric spots on the parotoids and the eyes (21.05% specimens) or they can be merged unilaterally (42.11% specimens) or bilaterally (36.84% specimens). The coral red colour can be limited to a thin colouration on the edge of the yellow spots or be the dominant background colour (Fig. 3). The specimens can show a completely black ventral region, or show some reddish colouration in the throat and / or the cloaca region. More rarely, they can show small white spots in the gular region (10.53% specimens). The only known specimen from the southernmost population (Sidi Ali) showed a predominantly black dorsal colouration, with five small yellow patches in the dorsum and seven on the tail (Fig. 3A). The size (TL) of adults ranges from 125.93–246.21 mm in males (*N* = 15, mean = 190.65 mm ± 7.74 standard error, SE) and 133.43–205.81 mm in females (*N* = 4, mean = 171.54 mm ± 16.67 SE). The differences in TL between both sexes were not significant (Mann-Whitney U test: *U* = 23, P = 0.484). Males mean SVL 93.71 mm ± 2.82 SE , HL 21.57 mm ± 0.76, PAL 12.81 mm ± 0.53, HW 15.87 mm ± 0.45, IOR 6.73 mm ± 0.25, ED 4.04 mm ± 0.12, END 4.69 mm ± 0.14, LHU 7.81 mm ± 0.32, FAL 9.57 mm ± 0.36, HAL 12.18 mm ± 0.56, THL 7.49 mm ± 0.31, TIL 10.26 mm ± 0.38, FL 15.34 mm ± 0.59. Females mean SVL 90.72 mm ± 8.33 SE, HL 20.18 mm ± 1.90, PAL 12.82 mm ± 1.11, HW 16.28 mm ± 1.33, IOR 7.33 mm ± 0.57, ED 3.71 ± 0.18, END 3.95 mm ± 0.39, LHU 7.28 mm ± 0.67, FAL 8.40 mm ± 0.86, HAL 11.73 mm ± 1.27, THL 7.36 mm ± 1.06, TIL 9.64 mm ± 1.10, FL 15.15 mm ± 2.69. Males have an ovoid-shaped cloaca during the breeding season and females a flat cloaca. Larvae are characterised by having the following morphological characters: Head depressed, with relatively large eyes situated laterally (Fig. 4). Snout rounded and semi-circular. Gills with three short rami and numerous fimbriae. Four fingers and five toes, narrow and pointed. Ten or eleven costal grooves. Tail equal in length to SVL or slightly smaller. Tail fin short, originating anterior to the pelvic girdle, ended in a bluntly pointed tip. Colour uniformly dark brown with a distinct pale spot at the base of the four limbs. The colouration varies according to the development and the terminal phases show diffuse yellow spots on the head and dorsum. SVL 29.0 mm, TAL 23.9 mm, HL 10.8, HW 6.2, TL 52.9 mm, MTH 5.6 mm (Taza, Morocco).

##### Distribution.

**Figure 5.**
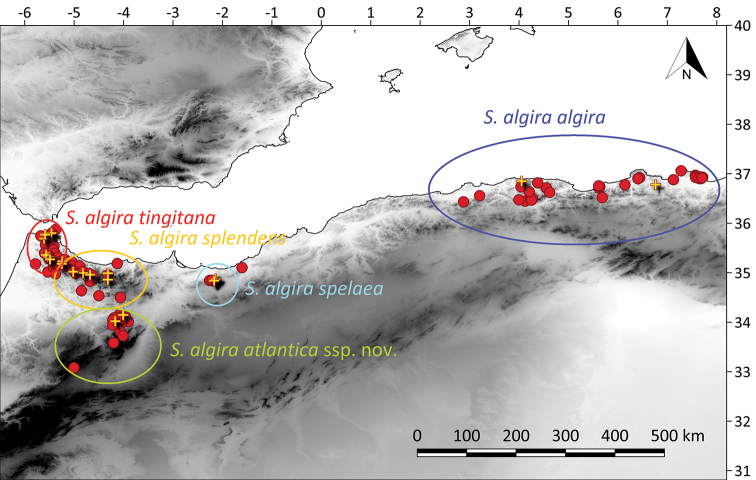
Map showing the distribution of the subspecies of *Salamandra algira* (red circles) and the localities included in the phylogenetic analyses (yellow crosses) in northern Morocco and Algeria.

*Salamandra algira atlantica* ssp. nov. is endemic to the northern and central Middle Atlas Mountains, northern Morocco: Tazekka National Park, Bou Iblane Massif and Jbel Sidi Ali (Fig. 5).

##### Natural history.

**Figure 6.**
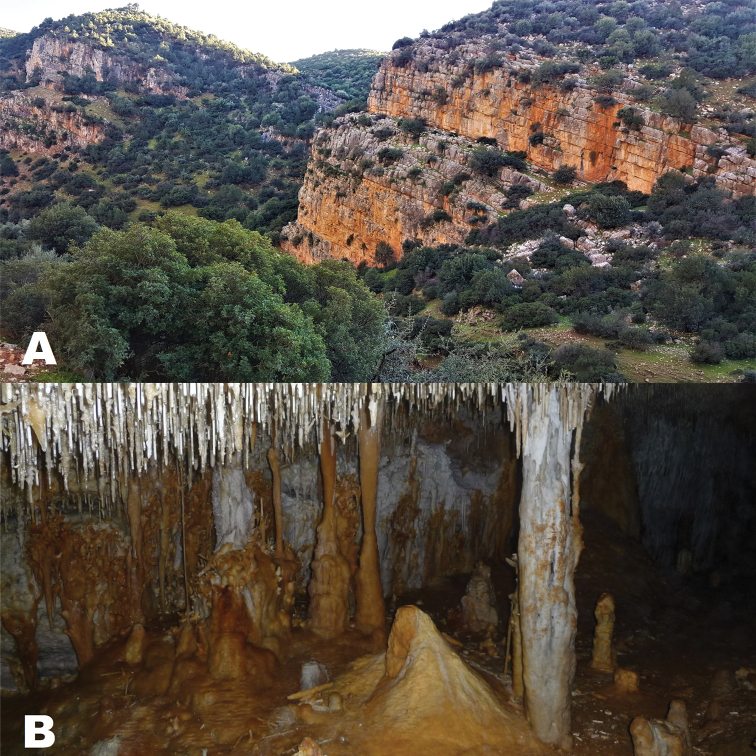
**A** Habitat of *Salamandra algira atlantica* ssp. nov.at the type locality, Taza Province, Middle Atlas Mountains, northern Morocco **B** Cave habitat at Grotte de Chaâra. Photographs by Axel Hernandez.

The new subspecies is found from 600–2455 m a.s.l. near springs and streams in humid mesothermal forests of conifers (*Abies pinsapo*, *Cedrus atlantica*, *Pinus halepensis*) and oaks (*Quercus ilex*, *Q. canariensis*, *Q. suber*; Fig. 6 A). There are also troglophile populations at Grotte de Chaâra, Grotte d’Izora. and Gouffre du Friouato which reproduce inside the caves at 400 m from the entrance (Fig. 6B). It is a crepuscular and nocturnal species having a surface activity from autumn to spring.

## Discussion

*Salamandra algira* was briefly described as Salamandra maculosa var. algira by Bedriaga (1883). As the holotype is not known to exist, a neotype (NHMW 9251) was later designated from Jebel Edough near Bône (Annaba), Algeria by Eiselt (1958). *Salamandra algira* had been considered as a subspecies of *S. salamandra*, until its specific and species status was confirmed by molecular analysis (Veith 1994; Steinfartz et al. 2000). Donaire-Barroso and Bogaerts (2003) described *S. algira tingitana* from Jbel Musa (= Jabal Muse; Tingitana Peninsula, northwestern Morocco), and subsequently *S. algira* was divided into two subspecies by separation of *S. algira tingitana* from the nomitypic *S. algira algira* from Annaba, Algeria. Escoriza et al. (2006) rediscovered a small and isolated population of *S. algira* in the Beni Snassen Massif (north-eastern Morocco). This population was shortly after described as a distinct and micro-endemic subspecies: *S. algira spelaea* (Escoriza and Comas 2007). In 2013, a fourth subspecies, *S. algira splendens* was described from the Rif and Middle Atlas Mountains. The type locality of *S. algira splendens* is Aïn Tissimilan, Jebel el Kelaâ, Chefchaouen, western Rif Mountains (Beukema et al. 2013).

As previously found (Escoriza et al. 2006; Dinis et al. 2019), our phylogenetic analysis recovered two main lineages within *S. algira*, one located west of the Moulouya valley, including the western subspecies (*S. algira tingitana* and *S. algira splendens*) and a second lineage formed by the eastern subspecies (*S. algira algira* and *S. algira spelaea*). Our results confirm that Algerian populations of *S. algira* are genetically distinct from the Moroccan ones (Ben Hassine et al. 2016). Additionally, our phylogenetic analysis corroborates the recent recognition of two main independent lineages in *S. algira tingitana* (Beukema et al. 2010, 2013). These different clades are mostly separated across the boundaries of river Martil, differing at the morphological level and by having two different reproduction modes, viviparous and larviparous (Beukema et al. 2010). The viviparous form of *S. algira tingitana*, which is only distributed in the extreme northern parts of the Tingitana Peninsula, was previously considered as a full valid species by Dubois and Raffaëlli (2009). This consideration is still under debate, despite unanimous recognition of two very divergent phenotypes and genotypes (Beukema et al. 2010, 2013). Consequently, *S. algira tingitana* may warrant taxonomic revision including new phylogenetic and morphological studies.

The separation among allopatric populations over North African mountainous systems can be attributed to the Late Neogene aridification (Griffin 2002; Escoriza and Ben Hassine 2015). The split between both subspecies of the western group, *S. algira tingitana* and *S. algira splendens*, was estimated during the Pliocene, approximately 1.6‒3.8 million years ago (Mya) while between the eastern subspecies, *S. algira algira* and *S. algira spelaea*, was estimated during the Plio-Pleistocene boundary, approximately 2.5 Mya (Beukema et al. 2010; Ben Hassine et al. 2016). *Salamandra algira atlantica* ssp. nov. is completely isolated from other populations of *S. algira* by the arid Moulouya and the Saïss plains. These contemporaneous arid barriers precluded the gene flow between Riffean and Middle Atlas populations and the Beni Snassen (Escoriza and Ben Hassine 2015).

Despite several attempts, the genus *Salamandra* still represents an outstanding and challenging complex due to conservative morphology, with a high degree of geographical, intraspecific, and genetic variation (Eiselt 1958; Steinfartz et al. 2000; García-París et al. 2003). The highest diversity is mainly found through the Iberian Peninsula, where nine subspecies are currently recognised (Joger and Steinfartz 1994; Veith 1994, 1996; Steinfartz et al. 2000). The recent classification and systematic arrangement are controversial and under debate (Thiesmeier 2004; Thiesmeier and Grossenbacher 2004; Dubois and Raffaëlli 2009; Raffaëlli 2013). Due to several discordance between nuclear and mtDNA gene trees usually found in previous studies, as a consequence of the retention of ancestral states, insufficient lineage sorting in the diverging populations or also resulting from gene introgression, their classification is difficult to assess (García-París et al. 2003), although *S. salamandra* sensu lato comprises several lineages representing three to four cryptic species (Raffaëlli 2013). In this sense, unique morphological traits, ecological differences and allopatric status, are crucial to evaluate properly new taxa among the genus *Salamandra* following recent descriptions (Malkmus 1983; Joger and Steinfartz 1994; Donaire-Barroso and Bogaerts 2003; Bonato and Steinfartz 2005; Köhler and Steinfartz 2006; Escoriza and Comas 2007; Beukema et al. 2013).

Our results indicated morphological divergence between *S. algira splendens* and *S. algira atlantica* ssp. nov. However, some specimens of this new subspecies are similar to *S. algira splendens*, thus their differentiation has to be supported by the geographical range. The presence of coral red colouration is evident in specimens of both subspecies, but is more extensive in some adult individuals of *S. algira atlantica* ssp. nov. In this sense, differences in the extension or presence of the red colouration was also considered as a diagnostical criterion for some Iberian subspecies: *S. salamandra bejarae*, *S. salamandra crespoi*, *S. salamandra gallaica* and *S. salamandra morenica* (Joger and Steinfartz 1994; Thiesmeier 2004). Reddish colouration is very scarce or absent in some species of the genus (e.g., *S. atra*, *S. corsica*) and several subspecies of *S. salamandra* (Thiesmeier 2004; Raffaëlli 2013; Sparreboom 2014).

In terms of conservation, *S. algira atlantica* ssp. nov. should be regarded as vulnerable. This endemic subspecies is distributed in a poorly prospected area of approximately 1600 km^2^, where it is almost exclusively confined to mid-high elevations. However, the distribution of the subspecies is still not completely understood: e.g., the southernmost population is only known from a single specimen and the hypogeal populations were also been discovered very recently (Hernandez 2018a, b, c). The alteration and destruction of natural habitats are the main threats found in the Middle Atlas Mountains.

## Conclusions

This study increases the current known number of subspecies of *S. algira* found in northern Morocco, from three (*S. algira tingitana*, *S. algira spelaea*, *S. algira splendens*) to four subspecies in describing *S. algira atlantica* ssp. nov. It also supports this region as having the highest intraspecific diversity of *S. algira*. The taxonomic separation of a single widespread species into multiple small-ranged taxa in turn have important implications for the conservation status of the original species. We therefore recommend a re-assessment of the outdated Vulnerable status (VU) of *S. algira* (IUCN 2009) to reflect the current taxonomic revisions and the increasing threats from the international pet trade and habitat loss which have taken place over the last decade.

## Supplementary Material

XML Treatment for Salamandra algira atlantica
